# Multiplex cytokine analysis of dermal interstitial blister fluid defines local disease mechanisms in systemic sclerosis

**DOI:** 10.1186/s13075-015-0575-8

**Published:** 2015-03-23

**Authors:** Kristina EN Clark, Henry Lopez, Bahja Ahmed Abdi, Sandra G Guerra, Xu Shiwen, Korsa Khan, Oseme Etomi, George R Martin, David J Abraham, Christopher P Denton, Richard J Stratton

**Affiliations:** Centre for Rheumatology and Connective Tissue Diseases, Royal Free Hospital Campus, University College Medical School, University College London, Rowland Hill Street, London, NW3 2PF UK; MuriGenics, Inc., 941 Railroad Avenue, Vallejo, CA 94592 USA; NIH, Institute of Ageing, MSC 2292, Bethesda, MD 20892 USA

## Abstract

**Introduction:**

Clinical diversity in systemic sclerosis (SSc) reflects multifaceted pathogenesis and the effect of key growth factors or cytokines operating within a disease-specific microenvironment. Dermal interstitial fluid sampling offers the potential to examine local mechanisms and identify proteins expressed within lesional tissue. We used multiplex cytokine analysis to profile the inflammatory and immune activity in the lesions of SSc patients.

**Methods:**

Dermal interstitial fluid sample from the involved forearm skin, and synchronous plasma samples were collected from SSc patients (n = 26, diffuse cutaneous SSc (DcSSc) n = 20, limited cutaneous SSc (LcSSc) n = 6), and healthy controls (HC) (n = 10) and profiled by Luminex® array for inflammatory cytokines, chemokines, and growth factors.

**Results:**

Luminex® profiling of the dermal blister fluid showed increased inflammatory cytokines (median interleukin ( IL)-6 in SSc 39.78 pg/ml, HC 5.51 pg/ml, p = 0.01, median IL-15 in SSc 6.27 pg/ml, HC 4.38 pg/ml, p = 0.03), chemokines (monocyte chemotactic protein (MCP)-3 9.81 pg/ml in SSc, 7.18 pg/ml HC, p = 0.04), and profibrotic growth factors (platelet derived growth factor (PDGF)-AA 10.38 pg/ml versus 6.94 pg/ml in HC, p = 0.03). In general dermal fluid and plasma cytokine levels did not correlate, consistent with predominantly local production of these factors within the dermal lesions, rather than leakage from the serum. In hierarchical clustering and network analysis IL-6 emerged as a key central mediator.

**Conclusions:**

Our data confirm that an immuno-inflammatory environment and aberrant vascular repair are intimately linked to fibroblast activation in lesional skin in SSc. This non-invasive method could be used to profile disease activity in the clinic, and identifies key inflammatory or pro-fibrotic proteins that might be targeted therapeutically. Distinct subgroups of SSc may be defined that show innate or adaptive immune cytokine signatures.

## Introduction

Clinical heterogeneity in systemic sclerosis (SSc) and variation in response to therapy is likely to reflect diverse underlying pathogenic mechanisms. Whole skin gene expression profiling has classified distinct molecular subsets with proliferative, fibrotic, limited and normal-like mRNA signatures [1] and associated these with possible treatment response to immunosuppression [2]. Response to therapy in SSc has traditionally been assessed by the modified Rodnan skin score [3], in addition to clinical and laboratory measures of organ function, and these have proven very valuable in clinical trials and pragmatic in routine clinical practice, but are not specifically linked to underlying biologic activity. Biomarker assays would allow for sensitive and reproducible means of targeting therapy and assessing disease response. Methods that define the molecular changes in lesional tissue of patients are needed and could link well with specific targeted treatments [4].

Gene expression biomarkers have been correlated with change in skin score [5] and, although not extensively studied, subset signatures have tended to be stable over time [1]. The aim of this study was to directly examine protein expression within the dermal interstitial fluid using a suction blister methodology [6], and to compare this with circulating levels.

The dermal suction blister method is a minimally invasive technique of sampling interstitial fluid. Søndergaard and colleagues developed the technique in SSc, noting increased levels of soluble intercellular adhesion molecule-1 and soluble interleukin (IL)-2 receptor in the interstitial fluid compared with the serum, mainly in the transition zone of the dermis [6].

Here we have used the novel approach of dermal suction blister fluid sampling combined with multiplex analysis to investigate protein expression within the dermal interstitial fluid in a well-characterised cohort of SSc patients and representative healthy controls (HCs). We have specifically compared the levels of key candidate proteins that may be relevant to pathogenesis or may reflect disease severity or activity. In addition we have compared the results for serum and dermal blister fluid samples within individual patients. This robust method has provided data that confirm the utility of this approach and highlighted potentially important aspects of disease biology. This approach could enable clinically useful biomarkers and allow application of targeted therapies to those most likely to benefit, based on the pattern of growth factor and cytokine expression.

## Methods

### Sample collection

Interstitial fluid samples from the forearm skin of patients with scleroderma and HCs were collected using the dermal suction blister method. The patients were all under the care of the Royal Free Hospital Centre for Rheumatology and Connective Tissue Diseases. All subjects provided informed consent, and this study was approved by the Local Research Ethics Committee (see Acknowledgements for details). The technique involved a dermal suction machine (Ventipress, Upsalla, Sweden) being adhered to newly sterilised forearm skin. The machine was attached to a clinic vacuum line and left for 2.5 hours, with suction pressure 280 mmHg through an 8 mm suction cup. Interstitial fluid was removed using a 23 gauge needle and stored in aliquots at −80°C. Between 100 and 250 μl blister fluid were collected from each patient. Plasma samples were obtained for each case, as close to the time of blister sampling as feasible. Plasma was aliquoted and frozen at −80°C for later analysis.

Clinical details on the patients were collected, which included type of disease, gender, age, date of disease onset, antibody status, current treatment, prior treatment, organ involvement, most recent forced vital capacity and forced expiratory volume in 1 second, recent creatinine, recent estimated pulmonary artery pressure on echocardiogram, and recent N-terminal brain natriuretic protein results. These data are all routinely tested on our cohort of patients as best practice.

### Sample analysis

Both the plasma and interstitial fluid samples were profiled by Luminex® (Life Technologies, Paisley, UK) bead array for inflammatory cytokines, chemokines, and growth factors including factors previously implicated in SSc pathogenesis (41 factors were analysed; see Tables 1 and 2). One aliquot of plasma and one aliquot of serum were analysed per subject.

**Table 1:** **Growth factor and cytokine profiling in systemic sclerosis and control blister fluid samples**

**Biologic function**		**Healthy control samples**	**SSc samples**	***P*** **value**
Innate immunity	IFNα2	5.34 (4.4 to 7.61)	6.35 (4.81 to 7.52)	0.35
	IL-1α	74.77 (22.48 to 132.28)	40.42 (15.27 to 113.19)	0.44
	IL-1B	0.37 (0.37 to 1.62)	0.62 (0.37 to 1.62)	0.57
	IL-1RA	802.5 (469.25 to 1,796.5)	695 (499.5 to 1,075.5)	0.72
	**IL-6**	**5.51 (2.44 to 26.83)**	**38.78 (19.23 to 88.81)**	**0.01**
	IL-12p40	5.16 (4.39 to 6.11)	4.58 (4.39 to 7.6)	0.86
	IL-12p70	2.03 (1.74 to 2.21)	1.99 (1.72 to 2.34)	0.87
	**IL-15**	**4.38 (3.2 to 4.95)**	**6.27 (4.21 to 9.34)**	**0.03**
	IP-10	988.5 (510.5 to 1,172.25)	1054 (547.25 to 1,929)	0.44
	TNFα	25.95 (12.15 to 65.36)	22.19 (14.46 to 44.1)	0.92
Adaptive immunity	IFNγ	1.23 (1.12 to 2.76)	1.58 (1.33 to 2.76)	0.19
	IL-2	1.26 (0.73 to 1.26)	1.26 (0.64 to 1.31)	0.57
	IL-3	1.62 (1.14 to 1.62)	1.62 (1.1 to 1.62)	0.78
	IL-4	2.6 (1.72 to 5.24)	3.955 (2.18 to 5.24)	0.54
	IL-5	1.28 (0.84 to 1.72)	0.96 (0.84 to 1.72)	0.83
	IL-7	4.16 (2.99 to 5.59)	4.89 (3.52 to 7.94)	0.13
	IL-9	0.77 (0.77 to 1.94)	0.77 (0.77 to 1.94)	0.86
	IL-10	22.02 (16.77 to 40.28)	33.05 (12.91 to 44.83)	0.83
	IL-13	1.95 (1.67 to 2.8)	1.98 (1.66 to 2.8)	0.81
	IL-17a	1.23 (1.23 to 1.34)	1.23 (1.23 to 1.34)	0.78
	sCD40L	284.5 (207.5 to 466.5)	285 (204.75 to 483)	0.83
	TNFβ	1.04 (1.04 to 3.95)	1.04 (1.04 to 3.95)	0.97
Chemokines	Eotaxin	37.95 (21.05 to 48.53)	31.46 (26.64 to 48.93)	0.92
	Fractalkine	37.96 (30.03 to 52.69)	39.61 (22.83 to 48.54)	0.62
	GRO	69.91 (47.16 to 252)	129 (78.92 to 246.25)	0.31
	IL-8	61.85 (28.05 to 234.75)	51.69 (37.24 to 80.03)	0.66
	MCP-1	762.5 (544 to 1,661.25)	792 (668.75 to 1,340.5)	0.92
	**MCP-3**	**7.18 (5.7 to 9.81)**	**9.81 (7.5 to 10.66)**	**0.04**
	MDC	1004.5 (715 to 1,126.75)	695 (554.5 to 867.25)	0.10
	MIP-1α	21.21 (3.51 to 47.18)	10.5 (3.51 to 16.46)	0.31
	MIP-1β	43.44 (8.13 to 111.17)	24.42 (12.89 to 45.65)	0.46
	RANTES	47.21 (19.68 to 178.01)	55.76 (33.4 to 101.35)	0.71
Growth factors	EGF	2.72 (2.72 to 10.12)	2.72 (2.72 to 7.25)	0.58
	Flt-3 L	70.25 (4.16 to 82.49)	51.82 (23.89 to 77.59)	0.45
	GCSF	3.26 (3.24 to 16.04)	3.24 (3.23 to 12.33)	0.69
	GMCSF	2.79 (1.88 to 9.33)	2.22 (1.74 to 7.24)	0.63
	**PDGF-AA**	**6.94 (5.87 to 7.78)**	**10.38 (7.4 to 17.71)**	**0.03**
	PDFG-BB	1.79 (0.41 to 1.79)	1.79 (1.79 to 3.2)	0.23
	TGF-α	7.09 (6.64 to 8.21)	7.0 (5.62 to 9.1)	0.62
	FGF-2	13.4 (11.67 to 16.8)	16.92 (13.4 to 20.24)	0.09
	VEGF	11.3 (6.92 to 16.92)	11.67 (10.28 to 22.29)	0.47

Data presented as median concentration (pg/ml) (25th to 75th percentile). Permutation analysis: significance analysis of microarrays for Excel, Wilcoxon rank-sum test. Significant results are in bold, taken as *P* <0.05. EGF, epidermal growth factor; FGF, fibroblast growth factor; Flt, FMS-like tyrosine kinase; GCSF, granulocyte colony-stimulating factor; GMCSF, granulocyte–macrophage colony-stimulating factor; GRO, growth regulated oncogene; IFN, interferon; IL, interleukin; IL-1RA, interleukin-1 receptor antagonist; IP-10, Interferon gamma induced protein 10; MCP, monocyte chemotactic protein; MIP, macrophage inflammatory protein; PDGF, platelet-derived growth factor; RANTES, regulated on activation normal T cell expressed and secreted; SSc, systemic sclerosis; TGF-α, transforming growth factor alpha; TNF-α, tumour necrosis factor alpha; VEGF, vascular endothelial cell growth factor.

**Table 2:** **Growth factor and cytokine profiling in systemic sclerosis and control plasma samples**

**Biologic function**		**Healthy control samples**	**SSc samples**	***P*** **value**
Innate immunity	IFNα2	20.16 (14.47 to 29.72)	28.86 (18.41 to 39.83)	0.33
	IL-1α	2.51 (2.51 to 5.78)	2.51 (2.51 to 17.91)	0.37
	IL-1B	1.65 (1.65 to 1.65)	1.65 (1.65 to 1.72)	0.40
	**IL-1RA**	**34.52 (25.15 to 44.60)**	**57.36 (35.85 to 83.9)**	**0.03**
	IL-6	2.66 (2.66 to 2.66)	2.66 (2.66 to 2.69)	0.29
	IL-12p40	4.77 (4.77 to 5.63)	12.92 (4.77 to 28.25)	0.07
	IL-12p70	3.78 (2.97 to 5.64)	4.73 (3.46 to 7.78)	0.34
	IL-15	2.45 (2.45 to 2.45)	2.45 (2.45 to 2.76)	0.29
	IP-10	297 (189.75 to 448)	396 (317.5 to 544.5)	0.27
	**TNFα**	**4.38 (3.98 to 6)**	**6.98 (4.98 to 8.84)**	**0.04**
Adaptive immunity	IFNγ	3.7 (2.99 to 4.49)	4.89 (3.26 to 6.82)	0.38
	IL-2	1.47 (1.47 to 1.47)	1.47 (1.47 to 2.14)	0.29
	IL-3	0.36 (0.34 to 0.54)	0.57 (0.34 to 0.82)	0.54
	IL-4	2.83 (2.83 to 2.83)	2.83 (2.83 to 3.65)	0.20
	IL-5	1.93 (1.93 to 1.93)	1.93 (1.93 to 1.93)	0.52
	IL-7	2.28 (1.78 to 2.89)	2.66 (2.14 to 3.85)	0.47
	IL-9	1.24 (1.24 to 1.24)	1.24 (1.24 to 1.24)	0.40
	IL-10	1.55 (1.03 to 2.91)	2.86 (1.48 to 5.18)	0.13
	IL-13	1.65 (1.65 to 1.65)	1.65 (1.65 to 1.65)	0.40
	IL-17α	2.33 (1.75 to 2.38)	2.49 (1.61 to 3.38)	0.38
	sCD40L	9,800.01 (9,800.01 to 9,800.01)	9,800.01 (9,800.01 to 9,800.01)	0.87
	TNFβ	2.34 (2.34 to 2.76)	2.34 (2.34 to 3.26)	0.49
Chemokines	Eotaxin	95.3 (67.43 to 129.75)	97.64 (76.3 to 132.5)	0.87
	Fractalkine	50.87 (41.87 to 57.36)	62.72 (49.39 to 72.85)	0.21
	GRO	627.5 (477.25 to 655)	556 (383.5 to 709)	0.52
	IL-8	3.11 (1.97 to 4.32)	3.29 (2.78 to 4.07)	0.58
	MCP-1	236.5 (217.5 to 240.5)	230 (157.5 to 274)	0.83
	MCP-3	8.84 (5.73 to 13.41)	14.68 (8.63 to 19.76)	0.06
	MDC	718 (686.75 to 785.5)	696 (624 to 835)	0.71
	MIP-1α	1.6 (1.6 to 2.03)	1.77 (1.6 to 3.99)	0.24
	MIP-1β	18.47 (16.15 to 24.73)	21.92 (17.75 to 27.85)	0.52
	**RANTES**	**1,869 (1,723.5 to 2,090.5)**	**1,473 (1,234 to 1,626)**	**0.01**
Growth factors	EGF	49.36 (38.36 to 144.75)	63.8 (47.29 to 128)	0.83
	Flt-3 L	5.07 (5.07 to 5.07)	5.07 (5.07 to 5.59)	0.29
	GCSF	35.64 (33.17 to 39.41)	43.21 (34.88 to 54.04)	0.21
	**GMCSF**	**7.9 (6.04 to 10.71)**	**12.84 (9.15 to 20.06)**	**0.05**
	PDGF-AA	1,518.5 (889 to 1,852)	1,300 (825.5 to 1,601.5)	0.49
	PDFG-BB	4,137 (2,686.5 to 5,063.75)	4,573 (3,646 to 5,609.5)	0.43
	TGFα	0.41 (0.41 to 0.41)	0.41 (0.41 to 0.41)	0.40
	FGF-2	59.52 (48.78 to 80.7)	78.75 (57.53 to 95.77)	0.12
	VEGF	71.78 (50.92 to 91.04)	130 (81.12 to 202)	0.08

Data presented as median concentration (pg/ml) (25th to 75th percentile). Permutation analysis: significance analysis of microarrays for Excel, Wilcoxon rank-sum test. Significant results are in bold, taken as *P* <0.05. EGF, epidermal growth factor; FGF, fibroblast growth factor; Flt, FMS-like tyrosine kinase; GCSF, granulocyte colony-stimulating factor; GMCSF, granulocyte–macrophage colony-stimulating factor; GRO, growth regulated oncogene; IFN, interferon; IL, interleukin; IL-1RA, interleukin-1 receptor antagonist; IP-10, Interferon gamma induced protein 10; MCP, monocyte chemotactic protein; MIP, macrophage inflammatory protein; PDGF, platelet-derived growth factor; RANTES, regulated on activation normal T cell expressed and secreted; SSc, systemic sclerosis; TGF-α, transforming growth factor alpha; TNF-α, tumour necrosis factor alpha; VEGF, vascular endothelial cell growth factor.

### Data analysis

Permutation analysis was used to compare cytokine levels in SSc and HC samples. This was processed in Excel (Microsoft, Redmond, Washington), and analysed using Wilcoxon rank-sum tests and significance analysis of microarray correction [7]. Where samples were undetectable below the threshold, the lowest detectable level was assigned; and where sample concentrations were greater than the range available for analysis, they were assigned the upper limit value of the range. Spearman’s rank correlation was used to assess any correlation between serum and interstitial fluid cytokine and growth factor levels. Hierarchical clustering and heat map construction was performed with CIMminer (Bethesda, Maryland, USA) using correlation to cluster both patients and protein factors. One-way analysis of variance with *post hoc* analysis was applied to the subgroup analysis derived from the hierarchical clustering.

### Network of potential protein interactions

Using the STRING 9.1 database (Swiss Institute of Bioinformatics, Lausanne, Switzerland), networks of potential protein interactions were created using the inflammatory proteins that were found to be significantly raised in the hierarchical clustering from our analysis, and were expanded to include downstream targets.

## Results

### Patients

In total, 26 patients with SSc were included in the analysis and 10 HCs. The clinical and laboratory features of the patients are presented in Table 3.

**Table 3:** **Demographic and clinical features of the study cohort**

**Disease characteristics**	**Systemic sclerosis patients**	**Healthy controls**
	**(** ***n*** **= 26)**	**(** ***n*** **= 10)**
Age (years)	55 ± 10	51 ± 14
Female sex	18 (69)	9 (90)
Diffuse cutaneous systemic sclerosis	20 (77)	
Disease duration (months)	9 ± 7.9	
Duration of Raynaud’s (months)	10 ± 1.6	
Modified Rodnan skin score	18.3 ± 11.24	
Organ involvement		
Oesophageal	69.2	
Other gastrointestinal	46.2	
Lung	42.3	
Muscle	30.8	
Joint	11.5	
Renal	3.8	
Cardiac	7.7	
Serology		
Anti-nuclear antibody positive	100	
Anti-RNA polymerase antibody	12	
Anti-topoisomerase antibody	38	
Anti-centromere antibody	15	
Other	35	

Data presented as mean ± standard deviation, *n* (%) or percentage.

The mean age of the SSc patients was 55 years (standard deviation (SD) 10). Eight SSc subjects were male and 18 were female. Twenty SSc patients were classified as diffuse cutaneous systemic sclerosis (DcSSc) and six had limited cutaneous systemic sclerosis (LcSSc). The mean duration of disease, defined by first non-Raynaud’s manifestation of SSc, was 9 years (SD 7.85), with a range from 1 to 33 years. Five patients had early disease, defined as disease duration <2 years. The mean modified Rodnan skin score of patients was 18.3 (SD 11.2). The mean age of control subjects was 51 years (SD 14). Nine HCs were female and one was male.

### Dermal blister fluid

The results of the interstitial fluid from the Luminex® array are presented in Table 1. This array identified seven cytokines whose mean concentrations were significantly higher in the patient cohort compared with the HC group. These cytokines included inflammatory cytokines (median IL-6: SSc 38.78 pg/ml, HC 5.51 pg/ml, *P* = 0.01; median IL-15: SSc 6.27 pg/ml, HC 4.38 pg/ml, *P* = 0.03), chemokines (monocyte chemotactic protein (MCP)-3: SSc 9.81 pg/ml, HC 7.18 pg/ml, *P* = 0.04), vascular growth factors (fibroblast growth factor (FGF)-2: SSc 16.92 pg/ml, HC 13.4 pg/ml, *P* = 0.09), and profibrotic growth factors (platelet-derived growth factor (PDGF)-AA: SSc 10.38 pg/ml, HC 6.94 pg/ml, *P* = 0.03) (factors grouped by biologic function summarised in Table 1) (significance analysis of microarray revealed these to have at least a 1.45-fold increase compared with controls).

Using Spearman rank correlation, there was no significant correlation between the significant proteins in the blister fluid and skin score or disease duration. MCP-1 was found to correlate negatively with disease duration (*r* = −0.406, *P* = 0.044). In addition we found that some factors were specific to the SSc dermal blister fluid samples and not detectable in HC samples. IL-17 was only detectable in DcSSc (5/19), and in 0/6 LcSSc and 0/10 HC. IL-6 showed a trend towards increased concentrations in DcSSc compared with LcSSc, but this was not statistically significant (mean 71.7 pg/ml in DcSSc, 32.3 pg/ml in LcSSc, *P* = 0.07). MCP-3 also showed a suggestion of concentrations in DcSSc compared with LcSSc, but again this was not statistically significant (mean 7.93 pg/ml in DcSSc, 6.09 pg/ml in LcSSc, *P* = 0.36).

### Plasma samples

Plasma samples were available from 19/26 SSc patients and 8/10 HCs, and these were included in the analysis. The results from the Luminex® array are presented in Table 2. Nine cytokines or chemokines were significantly raised in SSc patients compared with HCs. The proteins that were significantly raised in the serum (*P* <0.05) were IL-1 receptor antagonist (median: SSc 57.36 pg/ml, HC 34.52 pg/ml, *P* = 0.03), tumour necrosis factor alpha (median: SSc 6.98 pg/ml, HC 4.38 pg/ml, *P* = 0.04), regulated on activation, normal T cell expressed and secreted (median: SSc 1,473 pg/ml, HC 1,869 pg/ml, *P* = 0.01), and granulocyte–macrophage colony-stimulating factor (median: SSc 12.84 pg/ml, HC 7.9 pg/ml, *P* = 0.05). Using significant analysis of microarrays, MCP-3, IL-12p40, vascular endothelial growth factor, IL-10, IL-4, IL-2 and IL-1a were all found to have a greater than 1.5-fold increase in concentration compared with HCs.

Of interest, T-helper type (Th) 2 cytokines IL-4, IL-5 and IL-13 were only detectable in SSc plasma, although none of these were significant. IL-6 was also only detectable in SSc plasma, and not within HC plasma samples, but again was not significant (*P* = 0.07).

In total, 19/26 SSc patients had adequate samples for paired analysis. Comparing the Luminex® array profiling of the dermal blister fluid from those patients with SSc with the paired serum samples, none of the correlations reached significance. Other proinflammatory cytokines (IL-6: *r*^2^ = 0.083, *P* = 0.23; IL-17: *r*^2^ = 0.02, *P* = 0.56) and growth factors (vascular endothelial cell growth factor: *r*^2^ = 0.03, *P* = 0.47; PDGF: *r*^2^ = 0.08, *P* = 0.22) showed no correlation between concentrations in the serum samples and in the dermal blister fluid. This supports the notion that profiling the dermal interstitial fluid reflects the local inflammatory profile, as opposed to leakage from the serum [6].

**Figure 1:**
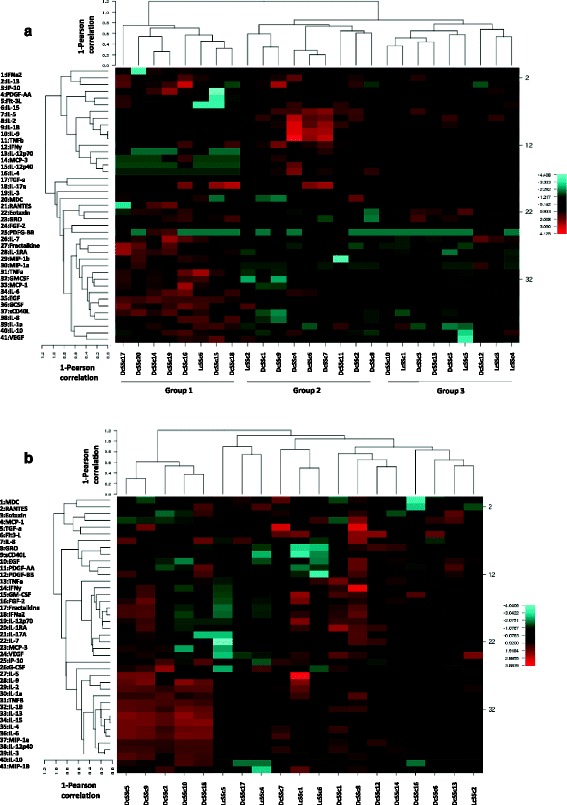
**Hierarchical clustering of plasma and blister fluid samples.** Heat maps of blister fluid **(a)** and plasma **(b)** for systemic sclerosis (SSc) patients characterised as limited cutaneous systemic sclerosis (LcSSc) or diffuse cutaneous systemic sclerosis (DcSSc). Blister fluid analysis was associated with clustering of SSc patients into three groups; Group 1, mainly DcSSc and characterised as interleukin (IL)-6, IL-10, tumour necrosis factor alpha (TNFα), and IL-1α high (innate inflammatory); Group 2, mainly DcSSc, and interferon gamma (IFNγ), IL-2, IL-4, IL-5, monocyte chemotactic protein (MCP)-3, IL-12p40 and IL-12p70 high (T lymphocyte, adaptive inflammatory); Group3, LcSSc and DcSSc, low levels of cytokines and chemokines (quiescent). Plasma analysis did not clearly cluster and in general did not correlate with the blister fluid analysis. EGF, epidermal growth factor; FGF, fibroblast growth factor; GCSF, granulocyte colony-stimulating factor; GMCSF, granulocyte–macrophage colony-stimulating factor; IL-1RA, interleukin-1 receptor antagonist, MIP, macrophage inflammatory protein; PDGF, platelet-derived growth factor; RANTES, regulated on activation normal T cell expressed and secreted; TGFβ, transforming growth factor beta; VEGF, vascular endothelial cell growth factor.

The HCs showed greater correlation between the Luminex® array results from the dermal blister fluid and serum samples, with many reaching significance. These included FGF-2 (*r*^2^ = 0.58, *P* = 0.03) and regulated on activation normal T cell expressed and secreted (*r*^2^ = 0.60, *P* = 0.02). Hierarchical clustering of plasma and blister fluid samples is shown in Figure 1.

### Hierarchical clustering

Protein concentrations were expressed as ratios of the mean value, log transformed and then expressed as SD above or below the mean value. Heat maps were then constructed for BF and plasma Luminex® results using CIMminer NIH software. Unbiased clustering was performed using correlation and the Pearson coefficient. The length of the dendrogram is inversely proportional to correlation. The resulting dendrogram shows clustering into three groups (Figure 1). Group 1 was characterised as IL-6, IL-10, tumour necrosis factor alpha and IL-1α high (innate inflammatory IL-6 associated), Group 2 by increased concentrations of interferon gamma (IFNγ), IL-2, IL-4, IL-5, MCP-3, IL12p40, IL12p70 (IFNγ Th cell group), and Group 3 by low levels of cytokines and chemokines (quiescent group). Plasma hierarchical clustering did not correlate with the blister fluid analysis (Figure 1b). A group of diffuse SSc patients was found to cluster in the plasma analysis but there was no correlation between this and the groups seen in blister fluid analysis.

Analysis of the clinical and laboratory characteristics of the patients is presented in Table 4, showing Group 1 to be early DcSSc with higher skin scores, Group 2 to be late-stage DcSSc, and that Group 3 contained LcSSc or DcSSc with low skin score. There was no apparent difference in antibody profiles, internal organ involvement or immunotherapy between the three groups. Matched plasma and blister fluid samples did not cluster together for individual patients (data not shown).

**Table 4:** **Patient characteristic analysis according to blister fluid heat map clusters**

**Group by heat map clustering**	**Diffuse/limited patient**	**Disease duration (years)**	**Skin score**	**Antibodies**	**Current therapy**	**Past therapy**	**Current steroid**
**Group 1**	DcSSc17	8	24	fs, RNApol	HCQ		No
Interleukin-6	DcSSc20	1	27	nuc	MMF		Yes
	DcSSc14	1	38	ACA	MMF		Yes
	DcSSc19	5	28	fs, U3RNP	HCQ	Campath1	No
	DcSSc16	2	34	hom, Scl70	MTX	CYC	Yes
	LcSSc6	3	6	ACA			No
	DcSSc15	5	35	hom, Scl70 + Ro	MMF		Yes
	DcSSc18	15	24	hom, Scl70	HCQ		No
Mean (SEM)		5 (1.7)	27 (3.5)				
**Group 2**	LcSSc2	23	6	fs, Scl70 + Ro	HCQ, MMF		Yes
Interferon gamma	DcSSc1	10	16	crs, nRNP + Ro	MMF, MTX	CYC	Yes
	DcSSc9	33	28	nuc	AZA	MTX, MMF, CIC	Yes
	DcSSc4	11	30	hom, Scl70	MMF, IMI	AZA, CYC	No
	DcSSc6	6	24	fs, RNA pol	MMF, HCQ	CYC	Yes
	DcSSc7	13	9	nuc,	HCQ		No
	DcSSc11	2	34	hom, Scl70	MTX	CYC	Yes
	DcSSc2	8	8	hom, Scl70	HCQ	MMF	No
	DcSSc8	20	30	hom + nuc, Scl70			No
Mean (SEM)		14 (3.2)	21 (3.6)				
**Group 3**	DcSSc10	3	12	nuc	IVIG	MMF	Yes
Quiescent	LcSSc1	6	4	ACA			No
	DcSSc3	8	15	fs, RNA pol	MTX	MMF	Yes
	DcSSc13	2	12	fs + cyto	MTX		Yes
	DcSSc5	20	10	hom, Scl70	MMF	CYC	Yes
	LcSSc5	12	15	fs			No
	DcSSc12	10	14	hom, Scl70	AZA	CYC	No
	LcSSc3	3	6	ACA			No
	LcSSc4	4	6	fs + nuc	MTX		Yes
Mean (SEM)		7.5 (1.9)	10 (1.4)				

Patients were grouped according to blister fluid heat map cluster. Patients in Group 1 (interleukin-6 high, innate inflammatory) were more likely to have early DcSSc with high skin score, Group 2 (interferon gamma high, effector T cell) were mainly late stage DcSSc, and Group 3 (quiescent pattern) were more likely to be LcSSc or DcSSc with low skin scores. Analysis of variance showed significant difference for disease duration between groups (*P* = 0.042) and skin score (*P* = 0.003). Scheffe *post hoc* analysis showed higher skin score in Group 1 compared with Group 3 (*P* = 0.005), otherwise *P* = not significant. Pattern of anti-nuclear antibody staining: homogeneous (hom), fine speckled (fs), nucleolar (nuc), coarse speckled (crs), cytoplasmic pattern (cyto). Therapy: azathioprine (AZA), cyclophosphamide (CYC), cyclosporine (CIC), hydroxychloroquine (HCQ), intravenous immunoglobulin (IVIG), mycophenolate (MMF), methotrexate (MTX). ACA, anticentromere antibody; DcSSc, diffuse cutaneous systemic sclerosis; LcSSc, limited cutaneous systemic sclerosis; SEM, standard error of the mean.

### Network analysis

**Figure 2:**
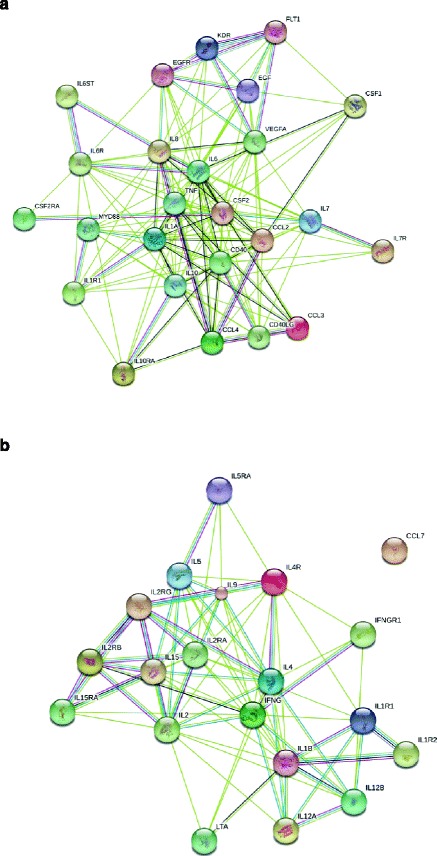
**Network string analysis derived from hierarchical clustering. (a)** Using the STRING 9.1 database, a network of potential protein interactions detected as increased in SSc dermal blister fluid of SSc patients clustered into Group 1. An innate inflammatory profile dominated by interleukin (IL)-6 and its associated interactions, as well as regulatory IL-10, were found to be increased. **(b)** Network of proteins detected as increased in the dermal blister fluid of SSc patients clustered into Group 2. Interferon gamma (IFNγ) and effector T-lymphocyte associated protein network predominates this subgroup. Light green, association by text mining; pink, association by experiment; black, association by co-expression; dark green, association by neighbourhood. Group 3 was quiescent (data not shown).

Using the STRING 9.1 database, a network of potential protein interactions was developed according to the subgroups derived from the hierarchical clustering (Figure 2a,b). Group 1 is characterised by IL-6 innate inflammatory protein pattern, with IL-6 occupying a central node with multiple downstream protein interactions. In Group 2 protein interactions, IFNγ appears as a central cytokine with multiple related immune and inflammatory proteins.

## Discussion

Skin involvement in SSc remains a challenging clinical problem in part because therapies need to be tested against reliable biomarkers of the local disease process. Our work has confirmed the feasibility of multiplex profiling of blister fluid, and overall the results are consistent with an inflammatory disease microenvironment in which T-cell responses as well as proangiogenic stimuli are being induced. The results of plasma analysis have shown a distinct pattern of systemic inflammation, which does not overlap with the blister fluid analysis. The blister fluid profiling may be giving an insight into the local pathological process within the local dermal tissue environment, as described previously by Søndergaard and colleagues [6]. SSc is a multisystem and multisite disease, and the results from the serum are likely to reflect this. The blister fluid allows us to profile the disease more locally, and allows for clearer interpretation of the data as we are only sampling from one organ. Søndergaard and colleagues have already shown that the blister fluid is acellular [6], and differences in albumin concentrations support our finding that the blister fluid is from the local environment and not drawn out from the plasma. More specifically, we found increased IL-6 in the blister fluid of our DcSSc patients, which supports earlier work by Khan and colleagues [8]. The hierarchical analysis of the blister fluid also identified a subgroup of patients who seem to have an innate inflammatory IL-6 associated profile within the dermis. Tocilizumab, a monoclonal antibody against IL-6, is currently undergoing trials as a potential targeted treatment in DcSSc patients with evidence of significantly affected skin disease, estimated using the modified Rodnan skin score and raised platelets. This method of profiling patients may help to aid identification of those who are likely to be more responsive to this treatment.

Downstream of IL-6, and in the presence of profibrotic transforming growth factor beta, Th17 responses are induced and are now believed to be important in the development of autoimmune inflammatory disorders including SSc [9]. Anti-IL-17 therapy has now been shown of benefit in some conditions, including psoriasis and ankylosing spondylitis [10,11]. However, inflammatory bowel disease was seen to worsen following anti-IL-17 consistent with a gut-specific protective role for IL-17 [12]. We found that IL-17, which was undetectable in HC samples, was induced in a subset of diffuse SSc patients, consistent with local Th17 polarisation. However, how important IL-17 is in promoting fibrosis and inflammation in SSc, and whether this subset of patients would benefit from anti-IL-17 targeted therapy, remain to be clarified. Several candidate treatment approaches in SSc are predicted to modulate Th17 function (halofuginone, tocilizumab) and agents targeting this factor are already in clinical use for psoriasis.

FGF-2 is an angiogenic factor induced by hypoxia, which usually results in increased angiogenesis [13]. We found that FGF-2 was increased in the SSc blister fluid, compared with HCs. There is a paradox in SSc where angiogenic factors are overexpressed, but the normal vascular repair seen in response to hypoxia and damage does not occur. FGF-2 could be involved in the deregulated vascular repair seen in SSc.

The results also support a role for PDGF in the SSc skin lesions where this factor may be acting as a chemoattractant and mitogen for fibroblasts. Clinical trials inhibiting PDGF tyrosine kinase receptor with imatinib have not been conclusive regarding whether it is PDGF that recruits fibroblasts to the activated areas of skin, but again patients could be stratified based on the dermal level of this factor [14].

The concentration of MCP-3 was also significantly increased in SSc blister fluid compared with HCs. This chemokine has previously been identified as a key mediator in skin fibrosis in SSc, and in the type 1 tight-skin mouse [15], and also correlated with the extent of skin sclerosis and the severity of lung fibrosis [16]. We did not find any correlation between MCP-3 concentrations and skin score, which is a finding previously noted from serum samples [16].

Limitations of this study include the low numbers of patients studied from disease subsets, and the limited number of proteins studied as well as their pleotropic nature. However, an attempt has been made to cluster patients into pathogenic groups based on the profile of blister fluid protein expression. Using an unbiased approach three groups were identified, and then protein–protein interaction software was used to describe the pathogenic pathways in the active groups, showing an IL-6-dominated inflammatory group, an IFNγ-associated inflammatory group as well as a quiescent group. These results have some parallel with the recent literature where gene expression profiling of skin biopsy material has been used to define mechanistic subgroups [17]. Of interest, the IFNγ inflammatory signature was seen in the blister fluid of some patients with very longstanding disease, and despite immunosuppressive therapy. In a recent elegant study, Gerber and colleagues have shown that primary abnormalities of the extracellular matrix can lead to inflammation and scleroderma-like autoimmunity [18]. It is possible that the abnormalities of the extracellular matrix are maintaining inflammatory and autoimmune responses in late stage SSc.

Our work supports the idea of profiling patients early in their disease via this minimally invasive technique. This dermal suction blister method could be used to target therapy towards the inflammatory profile of each patient. If combined with longitudinal assessment, the method could prove useful to predict disease progression and organ involvement depending on the inflammatory profile of the fluid, as well as response to treatments.

Potential biomarkers are under investigation to help predict severity of disease and organ involvement [19]. The dermal suction blister method offers a unique opportunity to complement biomarker research, by helping to discern whether the inflammatory profile is driven by local factors in the skin or systemic effects.

## Conclusions

Taken together, our findings confirm the feasibility and potential utility of dermal blister fluid to define local biological processes in SSc, and to identify profibrotic, angiogenic and T-cell-derived factors expressed locally within the skin lesions. In contrast, analysis of plasma samples revealed elevation of monocyte-derived inflammatory proteins. Discordance between the interstitial fluid samples and paired serum samples suggests that the dermal blister sampling method reflects local dermal protein expression. This dermal suction method offers an opportunity to profile the local inflammatory process occurring within the skin and has potential to complement clinical and gene expression-based classification to facilitate targeted therapy, as well as providing potential markers of disease activity or indicators of early treatment effect in proof-of-mechanism studies.

## Abbreviations

DcSSc: diffuse cutaneous systemic sclerosis
FGF: fibroblast growth factor
HC: healthy control
IFNγ: interferon gamma
IL: interleukin
LcSSc: limited cutaneous systemic sclerosis
MCP: monocyte chemotactic protein
PDGF: platelet-derived growth factor
SD: standard deviation
SSc: systemic sclerosis
Th: T-helper type
